# Evaluation of chromosomal abnormalities and copy number variations in late trimester pregnancy using cordocentesis

**DOI:** 10.18632/aging.103575

**Published:** 2020-08-15

**Authors:** Meiying Cai, Na Lin, Yuan Lin, Hailong Huang, Liangpu Xu

**Affiliations:** 1 Department of the Prenatal Diagnosis Center, Fujian Maternity and Child Health Hospital, Affiliated Hospital of Fujian Medical University, Fujian Key Laboratory for Prenatal Diagnosis and Birth Defect, Fuzhou, China

**Keywords:** cordocentesis, late trimester, SNP, cytogenetic abnormalities, sonographic anomalies

## Abstract

Because the numbers of detected fetal abnormalities increase as gestation progresses, we evaluated the safety and efficacy of cordocentesis for single nucleotide polymorphism (SNP) analysis tests in 754 women during third trimester pregnancy. Conventional karyotyping was performed on all fetuses, and Affymetrix CytoScan HD was used for SNP-array testing. In addition to the 24 cases with chromosomal abnormalities detected with conventional karyotyping analysis, the SNP-array test identified 56 (7.4%) cases with normal karyotypes but abnormal copy number variations (CNVs). Of those, 24 were pathogenic CNVs and 32 were of uncertain clinical significance. In 742 of the cases, there were abnormal sonographic findings, and cytogenetic abnormalities were detected in 76 cases (10.2%). The largest number of abnormalities involved multiple malformations (21.7%), followed by defects in the lymphatics or effusion (19.0%) or urogenital system (15.3%). The use of SNP-array test fully complemented chromosome karyotype analysis after late cordocentesis. It also improved the detection rate for fetal chromosomal abnormalities and was effective for preventing and controlling the occurrence of birth defects.

## INTRODUCTION

With the development of ultrasonic techniques, increasing numbers of abnormal fetuses are being found in the second and third trimesters of pregnancy. However, many pregnant women miss early and middle prenatal diagnoses for personal reasons, and the villi and amniotic fluid cells age as pregnancy progresses. This limits their availability for prenatal diagnosis. Therefore, prenatal diagnosis during third pregnancy requires cordocentesis, a technique entailing puncture of the umbilical vein through the mother's abdomen [1, 2].

Cordocentesis was developed during the 1980s and has greatly improved the success rate and accuracy of prenatal diagnosis. This has enabled expansion of diagnostic opportunities. Advantages of this technique are that it is not limited by gestation time, and that analysis of umbilical cord blood requires only short culture times and a simple chromosomal preparation procedure. Consequently, cordocentesis has become an irreplaceable and effective method of prenatal diagnosis in high-risk third trimester pregnancies. However, cordocentesis is more difficult and technically demanding than amniocentesis, with a higher rate of fetal loss. Thus, the choice between cordocentesis and amniocentesis should be based on weeks of gestation, fetal development, puncture technique, and laboratory capability.

Although acquired late during gestation, genetic results may relieve a pregnant woman’s anxieties if no cytogenetic abnormalities are found. Only a limited number of published reports have focused on fetal genetic information during third trimester pregnancies [3–5]. In the present study, we used cordocentesis and genome scanning with a SNP-array test and conventional karyotyping to analyze rates of cytogenetic abnormalities among third trimester pregnancies.

## RESULTS

**Figure 1:**
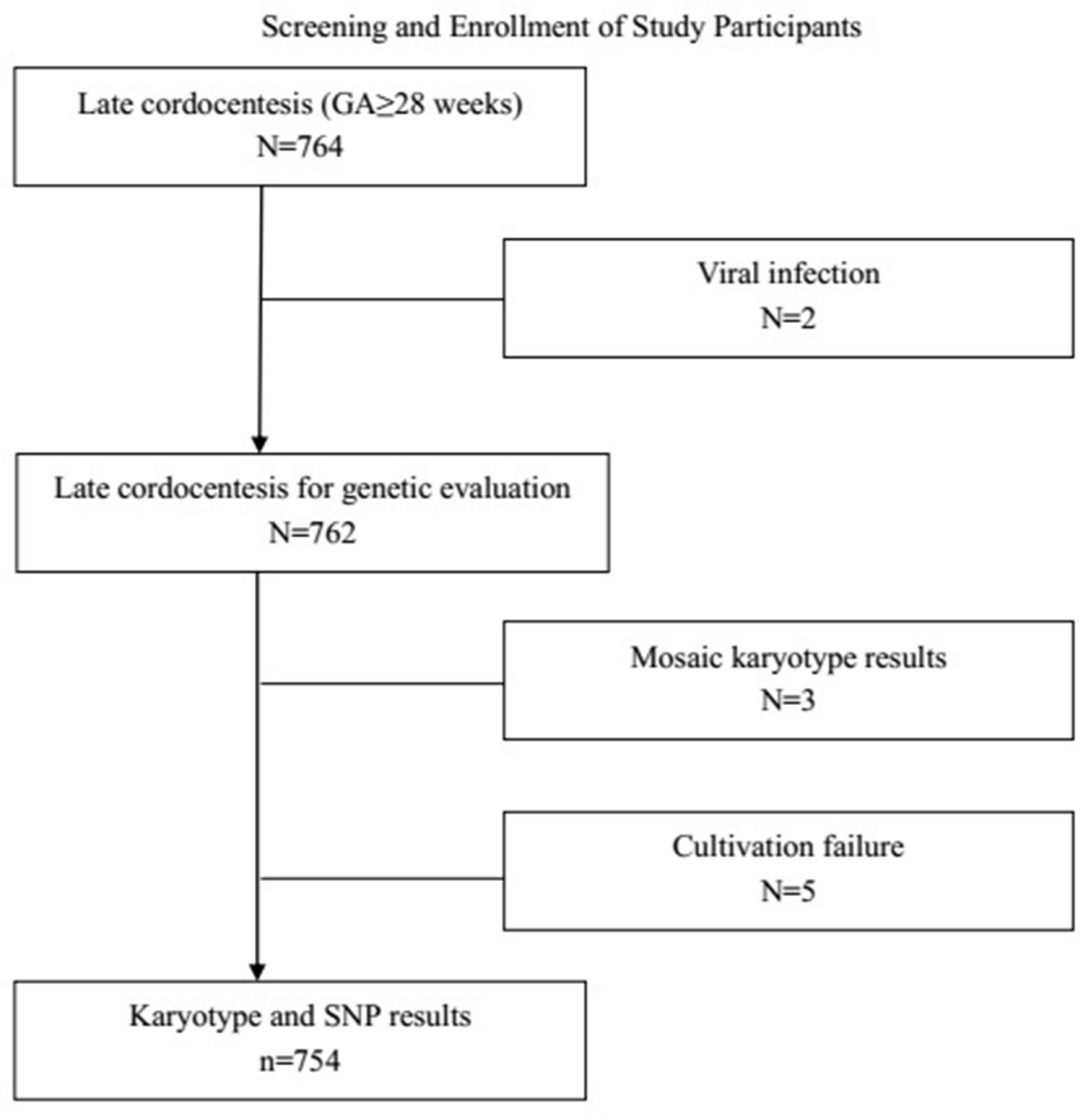
**Study participants were selected from all women at or beyond 28 gestational weeks who underwent amniocentesis at the prenatal diagnosis center between July 2017 and October 2019. GA: Gestationa age.**

Excluding two cases of viral infection, five cases in which there were failures of culture (poor cell growth that prevented karyotype analysis), and three cases with mosaic karyotype results, a combined total of 754 prenatal samples underwent SNP-array tests and conventional karyotyping (Figure 1). Of those, cytogenetic abnormalities were detected in 33.3% of cases that were positive on noninvasive prenatal testing (NIPT) and 10.2% of cases where anomalies were detected by ultrasonography (detailed information in Table 1).

**Table 1:** Phenotypic characteristics of 754 fetuses.

**Indication for Prenatal Diagnosis**	**Number**	**Number of cytogenetic abnormalities**	**Total (%)**
Anomaly on ultrasonography	742	76	10.2
Positive on NIPT	12	4	33.3

NIPT: noninvasive prenatal testing.

### Conventional karyotyping combined with SNP-array test

Conventional karyotyping analysis and SNP-array testing were performed with samples from all 754women included in the analysis. In addition to the 24 cases of chromosomal abnormalities that were also detected with conventional karyotyping analysis (Table 2), the SNP-array test identified 56 (7.4%) cases with normal karyotypes but abnormal copy number variations (CNVs) (Table 3). The number of abnormal CNVs detected with the SNP-array test was significantly higher than was detected with conventional karyotyping analysis (P <0.05).

**Table 2:** Abnormal karyotypes detected with conventional karyotyping analysis

**Case**	**Karyotype**	**Microarray nomenclature**	**Phenotypic characteristics of fetuses**	**Pathogenicity classification**	**Postnatal outcome**	**Inheritance**
1	47,XX,+21	arr[hg19](21) ×3	FGR, VSD	P	TP	*de novo*
2	47,XY,+21	arr[hg19](21) ×3	Absence of nasal bone	P	TP	*de novo*
3	47,XX,+21	arr[hg19](21) ×3	FGR,CHD	P	TP	*de novo*
4	47,XY,+21	arr[hg19](21) ×3	NIPT indicates high risk of trisomy 21	P	TP	*de novo*
5	47,XX,+13	arr[hg19](13)×3	CHD, Brain dysplasia, Orofacial clefts	P	TP	*de novo*
6	47, XXY	arr[hg19](1-22)×2, (XXY)×1	Hydronephrosis	P	TP	*de novo*
7	47, XXY	arr[hg19](1-22)×2,(XXY)×1	Echogenic bowel	P	TP	*de novo*
8	47,XXY	arr[hg19](1-22)×2,(XXY)×1	VSD, Echogenic bowel, FGR	P	TP	*de novo*
9	47,XXX	arr[hg19](X) ×3	FGR	P	TP	*de novo*
10	47, XY, +mar	arr[hg19]16P13.3(85,880-536,631)×1,17q24.2q25.3(64,966,574-81,041,823)×3	Ultrasound soft markers	P	TP	*de novo*
11	47, XY, +mar	arr[hg19]4q25q28.1(112,192,577-127,874,789)×1	Ultrasound soft markers	P	TP	*de novo*
12	46,XY,add16(p13.13)	arr[hg19]16p13.3(85,880-536,631)×1,17q24.2q25.3(64,966,574-81,041,823)×3	FGR	VOUS	TP	*de novo*
13	46,XX,add(12)(q24)	arr[hg19]11q23.2q25(113,998,447-134,937,416)×3,12q24.33(133,718,370-133,777,562) ×1	FGR	P	TP	*de novo*
14	46,XY,del(4)(p15)	arr[hg19]4p16.3 p15.1(68,345,431-35,252,743)×1	FGR, Nasal bone dysplasia	P	TP	*de novo*
15	46,XY,-21,+mar	arr[hg19]21q11.2q22.11(15,478,958-34,591,567) ×1,21q22.3(45,812,741-46,556,785) ×1,21q22.3(46,822,918-47,532,860) ×1	VSD, Bilateral ventricles widened	P	TP	*de novo*
16	45,X	arr[hg19](X) ×1	Posterior fossa widened	P	TP	*de novo*
17	46, XX, del(5)(p13)13p+	Arr[hg19]5p15.33p13.3(113,576-29, 220, 523) ×1	Dysplasia of corpus callosum	P	TP	*de novo*
18	47,XXX[51]/45,X[49]	arr[hg19](X)×1~2	NIPT indicates high risk of trisomy 13	P	TP	*de novo*
19	45,X[24]/46,XX[81]	arr[hg19](X) ×1-2	Positive on NIPT	P	TP	-
20	46,X,der(X)(Xqter→Xp22;: Xq21→Xqter)	arr[hg19]Xp22.33(168,551-2,958,480) ×1, Xq21.2q28(85,018,192-155,233,098) ×3,(XX) ×1	FGR	P	TP	*de novo*
21	46,XY, r i(18)(q10)[71]/ 46,XY, r idic(18)(p11q22)[6]/ 45,XY,-18 [3]	arr[hg19]18p11.32p11.31(136,227-6,010,824) ×1,18p11.31q23(6,010,999-74,605,367) ×3, 18q23 (75,171,951-78,013,728) ×1	Widened posterior fossa cistern, arachnoid cyst	P	TP	*de novo*
22	46,XX,r(18)(p11q22)[97]/46,XX,idic r(18)(p11q22)[13]/45,XX,-18[3]/47,XX,idic r(18)(p11q22)[2]	arr[hg19]18p11.32p11.31(136,227-3,334,683) ×1,18p11.31q22.3(3,342,699-72,722,952) ×3, 18q22.3q23 (72,723,195-78,013,728) ×1	NIPT indicates chromosome 18 is fully or partially missing	P	TP	*de novo*
23	46, XX, t(5;14)(p13.3;q21)14P+	arr[hg19]Xq28(152,446,333-153,581,657) ×3, 1p36.33p36.23(849,466-592,172) ×1, 1q44(246,015,892-249,224,684) ×3	Bilateral ventricles widened	P	TP	*de novo*
24	46,XY,r(p22q36)[86]/46,XY,dic r(7;7)(p22q36;p22q36)[4]	arr[hg19]7p22.3q36.1(43,376-149,349,749) ×2-3, 7q36.1q36.3(150,918,631-159,119,707) ×1	spinal malformation	P	TP	*de novo*

CHD: Congenital heart disease; FGR: fetal growth restriction; NIPT: noninvasive prenatal testing; P: pathogenic; TD: term delivery; TP: termination of pregnancy; VSD: ventricular septal defect.

### Detection rates of the SNP-array test with normal karyotype

Of the additional 56 cases with abnormal CNVs identified through SNP-array testing, 24 were pathogenic CNVs and 32 were of uncertain clinical significance (VUS). The pathogenic CNVs were associated with known chromosomal disorders, including 22q11 deletion syndrome, 17q12 deletion syndrome and 16p11.2 deletion syndrome. They were also related to deletions of 4p16.3 p16.1, 5q35.2q35.3, 7q11.2, 10q11.22q11.23, 17p12, 17p13.3p13.2 and 22q13.33; duplications of 3q29, 7q11.23, 15 q13.3 9q21.33q22.1 and 17p11.2; and uniparental disomy (UPD) in 15 q14 q21.3. The VUS CNVs were related to microdeletions ranging from 0.42Mb to 5.5Mb in length and microduplications varying from 0.68 Mb to1.5 Mb (Table 3).

**Table 3:** Chromosomal microarray analysis using SNP array testing of samples with normal karyotypes.

**Case**	**Microarray nomenclature**	**Size (Mb)**	**Phenotypic characteristics of fetuses**	**Pathogenicity classification**	**Obstetric outcomes**	**Inheritance**
1	arr[hg19]22q11.21 (18,916,842-21,800,471) ×1	2.9	CHD	P	TP	*de novo*
2	arr[hg19]22q11.21 (18,648,855-21,800,471) ×1	3.1	CHD	P	TP	*de novo*
3	arr[hg19]22q11.21 (18,648,855-21,800,471) ×1	3.1	CHD, thymic dysplasia	P	TP	*de novo*
4	arr[hg19]22q11.21 (18,649,189-21,800,471) ×1	3.1	CHD	P	TP	*de novo*
5	arr[hg19]22q11.21 (18,648,855-21,800,471) ×1	3.1	CHD	P	TP	*de novo*
6	arr[hg19]22q11.21 (20,730,143-21,800,471) ×1	1.0	Multiple cysts of left choroid plexus, left renal cysts, and varus	P	TP	*de novo*
7	arr[hg19]17q12 (34,822,465-36, 404, 555) ×1	1.58	Double kidney echo enhancement	P	TP	*de novo*
8	arr[hg19]17q12 (34,822,465-36, 243, 365) ×1	1.4	Double kidney echo enhancement	P	TP	*de novo*
9	arr[hg19]17q12 (34,822,465-36, 307, 773) ×1	1.48	Double kidney echo enhancement	P	TP	Maternal
10	arr[hg19]16p11.2 (28,810,324-29,032,280)×1	0.22	Lateral ventricle widens	P	TP	*de novo*
11	arr[hg19]16p11.2 (29,567,296-30,190,029)×1	0.6	Lateral ventricle widens	P	TP	*de novo*
12	arr[hg19]16p11.2 (29,591,326-30,176,508)×1	0.57	Hydrocephalus	P	TP	*de novo*
13	arr[hg19]3q29 (195,743,957-197,386,180) ×3	1.6	VSD	P	TP	*de novo*
14	arr[hg19]4p16.3 p16.1 (68,345-6,608,624)×1	6.5	CHD	P	TP	*de novo*
15	arr[hg19]5q35.2q35.3 (175,416,095-177,482, 506) ×1	2.0	Lateral ventricle widens	P	TP	*de novo*
16	arr[hg19]7q11.23 (72,723,370-74,143,240)×1	1.42	FGR	P	TP	*de novo*
17	arr[hg19]7q11.23 (72,701,098-74,069,645)×3	1.3	VSD, Unilateral renal agenesis	P	TP	*de novo*
18	arr[hg19]10q11.22q11.23 (46,252,072-51,903,756) ×1	5.6	FGR	P	TP	*de novo*
19	arr[hg19]15q14q21.3 (35,077,111-54,347,324)hmz	19.2	FGR	P	TP	UPD
20	arr[hg19]15 q13.3 (32,011,458-32,914,239)×3	0.88	Half vertebral body	P	TP	Paternal
21	arr[hg19]17p12 (14,083,054-15,482,833) ×1	1.4	Left renal dysplasia	P	TP	Maternal
22	arr[hg19]15q11.2 (22,770,421-23,277,436)×1	1.2	VSD, Brain dysplasia	P	TP	paternal
23	arr[hg19]17p13.3p13.2 (525-5,204,373)×1	5.2	Bilateral ventricles widened, cerebellum entricular dysplasia	P	TP	*de novo*
24	arr[hg19]22q13.33 (49,683,904-51,197,766) ×1	3.1	Echogenic bowel	P	TP	*de novo*
25	arr[hg19]16p13.11 (15,510,512-16,309,046)×3	0.78	Tricuspid regurgitation	VOUS	TD	-
26	arr[hg19]1q21.1 (145,375,770-145,770,627)×1,9p24.1 (4,623,660-5,501,699) ×3	0.68,0.86	Lateral ventricle widens	VOUS	TD	*de novo*
27	arr[hg19]1q21.1q21.2 (145,995,176-147,398,268)×3	1.4	CHD	VOUS	TP	*de novo*
28	arr[hg19]1q21.1q21.2 (145,958,361-147,830,830)×3	1.8	Lateral ventricle widens	VOUS	TD	*de novo*
29	arr[hg19]2q22.2 (143,043,284-143,866,399)×4	0.80	Effusion	VOUS	TD	*de novo*
30	arr[hg19]2q36.1q36.2 (224,459,152-225,330,583)×3	0.85	Posterior fossa widened	VOUS	TD	*de novo*
31	arr[hg19]2q11.1q11.2 (96,679,225-97,669,032)×1	0.97	Hydronephrosis	VOUS	TD	*de novo*
32	arr[hg19]3p26.3 (1,855,754-2,663,625)×1	0.79	Bilateral ventricles widened	VOUS	TD	-
33	arr[hg19]3q26.1q29(163,256,369-197,791,601)hmz,5p13.1p11 (41,029,137-46,313,469)hmz,6q24. 2q25(143,341,406-161,527,784) hmz,12q13.2q21.2(56,011,100-77,134,151)hmz,17q21.2q21.32(39,639,602-45,479,706)hmz,21q21. 3q22.2(28,124,165-42,352,287)hmz	99.1	Lateral ventricle widens	VOUS	TD	-
34	arr[hg19]3p22.1 (42,875,130-43,309,436)×1	0.42	Lateral ventricle widens	VOUS	TD	*de novo*
35	arr[hg19]3q28(188,788,120-191,331,505) ×1,15q11.2(23,620,191-24,978,547) ×3	2.5	Unilateral renal agenesis	VOUS	TD	*de novo*
36	arr[hg19]3p22.3 (33,805,560-35,318,562)×3	1.5	Effusion	VOUS	TD	Maternal
37	arr[hg19]5p15.33p15.31 (4,482,234-6,636,035)×1	0.61	Effusion	VOUS	TP	*de novo*
38	arr[hg19]4q28.3q31.3 (133,718,289-154,569,367)hmz	20.8	FGR	VOUS	TD	*de novo*
39	arr[hg19]4q24(106,284,925-107,545,257)×3	1.2	VSD	VOUS	TD	*de novo*
40	arr[hg19]8p23.2(3,703,883-5,940,433) ×3	2.2	Bilateral choroid plexus cysts	VOUS	TD	*de novo*
41	arr[hg19]10q24.31Q24.32 (102,972,457-103,179,063)×3	0.20	Posterior fossa widened	VOUS	TD	*de novo*
42	arr[hg19]10q11.21q11.22 (42,433,738-48,006,310) ×1	5.5	Echogenic bowel	VOUS	TD	*de novo*
43	arr[hg19]13q14.3 (52,649,105-53,172,866) ×3	0.53	Hydronephrosis	VOUS	TD	*de novo*
44	arr[hg19]14q21.2q21.3 (46,782,405-49,288,860) ×1	2.5	Hydrocephalus	VOUS	TP	*de novo*
45	arr[hg19]15q11.2 (22,770,421-23,082,237)×1	0.30	Ultrasound soft markers	VOUS	TD	*de novo*
46	arr[hg19]15q11.2 (22,770,421-23,286,423)×1	0.5	Ultrasound soft markers	VOUS	TD	Paternal
47	arr[hg19]15 q13.3 (31,999,631-32,444,043)×3	0.43	Severe hydrocephalus	VOUS	TP	*de novo*
48	arr[hg19]15 q13.3 (32,003,537-32,444,043)×3	0.43	CHD	VOUS	TP	*de novo*
49	arr[hg19]16p13.11 (15,325,072-16,272,403)×3	0.92	Bilateral hydronephrosis	VOUS	TP	*de novo*
50	arr[hg19]16p13.11 (15,171,146-16,309,046)×3	1.1	Echogenic bowel	VOUS	TD	Maternal
51	arr[hg19]16p13.11 (14,897,401-16,534,031)×1	1.6	VSD	VOUS	TD	*de novo*
52	arr[hg19]16p11.2 (29,580,020-30,190,029)×1	0.60	Spinal dysplasia	VOUS	TP	*de novo*
53	arr[hg19]17q21.31 (41,774,473-42,491,805)×4	0.70	FGR	VOUS	TD	*de novo*
54	arr[hg19]22q11.21 (18,648,855-21,459,713) ×3	2.8	CHD	VOUS	TP	Maternal
55	arr[hg19]22q11.21 (18,648,855-21,800,471) ×3	3.1	FGR	VOUS	TD	Paternal
56	arr[hg19]Xp21.1 (32,670,116-32,891,702) ×1	0.22	Effusion	VOUS	TD	*de novo*

CHD: Congenital heart disease; FGR: fetal growth restriction; NIPT noninvasive prenatal testing; P: pathogenic; TD: term delivery; TP: termination of pregnancy; UPD: uniparental disomy; VSD: ventricular septal defect.

### Karyotyping and SNP-array test findings in relation to sonographic anomalies

Of the 754 fetuses, 742 exhibited anomalies on ultrasonography. Cytogenetic abnormalities were detected in 10.2% of patients (n = 76/742). The greatest number were due to multiple malformations (21.7%), followed by abnormalities of the lymphatics or effusion (19.0%), urogenital system (15.3%), skeletal system (10.3%) and central nervous system (10.2%) (Table 4). No abnormalities of the digestive system, respiratory system or craniofacial region were detected.

**Table 4:** Frequency of abnormal sonographic findings (numbers of fetuses) (%).

**Ultrasound findings**	**Total**	**Number of cytogenetic abnormalities**		**(%)**
		**Abnormal karyotype**	**Abnormal CMA**	
Multiple malformations	46	6	4	21.7
Lymphatic or effusion	21	0	4	19.0
Urogenital system	59	1	8	15.3
Skeletal system	29	1	2	10.3
Central nervous system	177	3	15	10.2
Cardiovascular system	123	1	11	9.8
FGR	120	4	6	8.3
Ultrasound soft markers	121	4	6	8.3
Digestive system	25	0	0	0
Respiratory system	13	0	0	0
Craniofacial region	8	0	0	0

FGR: fetal growth restriction; CMA:chromosomal microarray analysis.

## DISCUSSION

Cordocentesis can be used in pregnancy after 17 weeks as a remedy when amniocentesis has failed. Blood cell cultures avoid the phenomenon of chromosome pseudo chimeras sometimes seen in amniotic fluid cell cultures. However, cordocentesis is riskier and more difficult to perform than amniocentesis, and should be conducted with the assistance of an experienced clinician and sonographer. Factors associated with fetal loss in cordocentesis include surgical proficiency, fetal structural defects (including fluid accumulation), intrauterine growth restriction and early gestation. In the present study, fetal loss due to cordocentesis was zero, lower than the risk of miscarriage (1-2%) reported by Ghi et al [6].

Using cordocentesis, we detected cytogenetic abnormalities in 10.6% (80/754) of fetuses in third trimester pregnancies. Karyotype analysis identified chromosomal abnormalities in 3.2% of the cases, which is fewer than in other reports [7, 8]. SNP-array tests detected cytogenetic abnormalities in an additional 7.4% of cases. This rate is slightly higher than the incidence of abnormal CNVs in the second trimester of pregnancy. The 24 cases in which karyotype analysis identified clinically significant chromosomal abnormalities included four cases of trisomy 21, three of Klinefelter's Syndrome and six of unbalanced translocations. Phenotypic ultrasound characteristics were normal during first and second pregnancy in these cases, but were abnormal in the third trimester. The majority of ultrasonographic abnormalities of these fetuses were cardiac malformations and fetal growth restriction. Ultrasonic examination is an important component of prenatal diagnosis. As gestation proceeds, ultrasound is capable of detecting additional fetal developmental or morphological abnormalities; that is, the incidences of fetal abnormality and growth restriction increase as gestation proceeds [9, 10]. Thus, although fetal malformation is often a manifestation of chromosomal abnormalities, many anatomical abnormalities are detected by ultrasound only in the third trimester. In such cases, it is important to perform third gestation cordocentesis. In addition, the occurrence of balanced translocations in four cases led us to detect abnormal karyotypes with normal SNP-array results. These cannot be detected using SNP-array tests because there is no gain or loss of genetic material. Genetically balanced rearrangement may have no effect on a current pregnancy, but may still be helpful for future reproductive counselling.

Among the 24 pathogenic CNVs in cases with normal karyotypes were six identified as deletions of 22q11.2 and three 17q12 microdeletions. 22q11.2 microdeletion syndrome [11] is known to be associated with congenital heart defects [12], while 17q12 microdeletion syndrome [13] is associated with congenital anomalies of the kidney and urinary tract [14]. These were the most frequently detected and confirmed anomalies in the present study. We also identified three fetuses with 16p11.2 deletion syndrome [15]. These fetuses exhibited central nervous system deformations, which ultrasonography showed to be lateral ventricle widening in two fetuses and hydrocephalus in one. Earlier reports suggest 16p11.2 deletion is associated with autism spectrum disorder [16] and schizophrenia [17, 18]. SNP-array results from the parents of these three fetuses revealed that the abnormalities were *de novo*. We classified the 16p11.2 deletion as a pathogenic variation. We also identified 10 cases of non-syndromic pathogenic CNVs, namely deletions of 4p16.3 p16.1, 5q35.2q35.3, 7q11.2, 10q11.22q11.23, 17p12, 17p13.3p13.2 and 22q13.33, and duplications of 3q29, 7q11.23 and 15q13.3. According to the International Standards for Cytogenomic Arrays (ISCA) and the Decipher databases, the detected variations at these loci are pathogenic. We found one fetus with fetal growth restriction accompanied by a loss of heterozygosity at 15 q14 q21.3. The parental SNP-array test revealed that this was a result of maternal uniparental disomy (UPD) 15, which causes Prader-Willi syndrome. According to published reports [19–21], UPD of Chr15 is a pathogenic CNV with phenotypes generally involving intrauterine growth retardation.

In the present study, the largest number of third trimester pregnancy cordocentesis were performed on women after fetal abnormality had been detected with ultrasonography. Bardin et al. [3] reported that 29.1% fetuses in which acentral nervous system abnormality was detected at or beyond 23 weeks of gestationhad at least one earlier normal anatomical examination. The most common late onset abnormal sonographic finding was ventriculomegaly. Similarly, Yinon et al. [10] reported that among those diagnosed with central nervous system abnormalities, 5.5% had late-onset abnormalities detected after at least one normal anatomical examination at no later than 19-23 weeks. The most common late onset abnormal sonographic findings involved intracranial cysts and ventriculomegaly. Consistent with these two cohorts, the most common late onset abnormal sonographic findings in the present study involved the central nervous system (177/742, 23.9%), with ventriculomegaly being the most frequent sonographic finding. In two large-scale retrospective studies, ultrasound anomalies were detected in between 6% and 8% of cases with a normal karyotype in the early and middle trimester of pregnancy [22, 23]. We therefore recommend that if cordocentesis is performed, a SNP-array test should be included as part of the genome analysis for fetuses in the third trimester of pregnancy.

The challenge of SNP-array tests in clinical practice is detection of large numbers of VUS. We observed VUS CNVs in 4.5% of cases. This is consistent with previously reported rates of VUS, which range from 0.39% to 4.2% [7, 23, 24]. Two reports [25, 26] have commented that the high heterogeneity of the results from different studies using microarray analyses may reflect multiple factors, including the types of samples studied and the detection platform used. The difference between VUS and pathogenic CNVs is that VUS CNVs are not present in an existing online public database, nor are they currently described in the literature. Additionally, no similar CNVs were found by SNP-array tests of the genomic DNA from the parents. Consequently, the clinical significance of CNVs is not yet clear. Given the limitations of the SNP-array test, we cannot rule out that VUS CNVs are caused by minor gene deletions/duplications or base mutations that are below the resolution of the existing SNP-array test platforms. Addressing that limitation with require increasingly advanced technology to be used in prenatal diagnosis [27–29].

In conclusion, we suggest that among the invasive prenatal diagnostic techniques, cordocentesis is safe and reliable, and fulfills the requirements for prenatal diagnosis of high-risk pregnant women late during gestation. The use of SNP-array tests can complement chromosome karyotype analysis to improve detection of fetal chromosomal abnormalities and control the occurrence of birth defects. We therefore suggest that prenatal diagnosis in third pregnancy is as important as it is in first and second pregnancy.

## MATERIALS AND METHODS

### Patient data

Between July 2017 and October 2019, 754 cordocentesis were performed after obtaining signed consent and providing appropriate counselling at the Prenatal Diagnosis Center of the Fujian Provincial Maternal and Children Health Hospital. Mean maternal age was 29.6 years (range: 19-46 years) and mean gestation at the time of cordocentesis was 29.8 weeks (range: 28-38 weeks). Inclusion criteria for the analysis were: (1) an ultrasonographic abnormality first detected and diagnosed in third trimester pregnancy, including fetal malformations in the skeletal system, urogenital system, central nervous system or cardiovascular system; fetal growth restriction (FGR); or ultrasound soft markers and (2) positive on noninvasive prenatal testing (NIPT) (detailed information in Table 1). The ethical conduct of this study was approved by the Fujian Provincial Maternal and Child Health Hospital.

### Conventional karyotyping

Cultured lymphocytes were analyzed by conventional karyotyping using Giemsa banding at a resolution of 450-550 bands.

### SNP-array test

Fetal blood (1mL) was collected by cordocentesis. Genomic DNA was extracted directly from the fetal blood using a QIAamp DNA Blood Mini kit (Qiagen). Short tandem repeats (STR) analysis was conducted before detecting of the fetal samples. SNP-array tests were conducted using a method previously established in our laboratory [30]. Procedures for all genomic DNA sample fragmentation, labeling, and array hybridization were performed in accordance with the manufacturer’s instructions. The CNV report filter was set to >100 kb in length, with a minimum of 50 tags. The results obtained from the CytoScan array scanning were analyzed using Chromosome Analysis Suite software (Affymetrix, Santa Clara, CA) and annotated based on genome version GRCh37 (hg19). CNVs were classified as being pathogenic, benign or of uncertain clinical significance (VUS) according to the American College of Medical Genetics (ACMG) guidelines [31]. Parental testing was performed for fetuses that had abnormal SNP-array results to determine the pattern of inheritance of copy number gains/losses. All annotated CNVs were verified by fluorescence in situ hybridization (FISH).

### Statistical analysis

Statistical analysis was performed using SPSS Statistics v20 software (IBM, Armonk, NY). Rates of detection of cytogenetic abnormalities in the fetuses using conventional karyotyping were compared with results from the SNP array. Chi-square test was used to compare the rates of detection. Values of P <0.05 were considered statistically significant.
